# 3-iodotyrosine fluorescent proteins for improved expansion microscopy

**DOI:** 10.17912/micropub.biology.002234

**Published:** 2026-07-21

**Authors:** Will Scott, Nimo Abdullah, Ragnheiður Guðjónsdóttir,, Teresa Massam-Wu, Maria Denisova, Corenna Smith, Falk Schneider, Karuna Sampath, Masanori Mishima, Mohan Balasubramanian

**Affiliations:** 1 Centre for Mechanochemical Cell Biology, Warwick Medical School, University of Warwick, University of Warwick, Coventry, ENG, United Kingdom; 2 Cellular Interfaces Cluster, Centre for Mechanochemical Cell Biology, Warwick Medical School, University of Warwick, University of Warwick, Coventry, ENG, United Kingdom; 3 Centre for Early Life, Warwick Medical School, University of Warwick, University of Warwick, Coventry, ENG, United Kingdom; 4 Centre for Mechanochemical Cell Biology, Division of Biomedical Sciences, Warwick Medical School, University of Warwick, University of Warwick, Coventry, ENG, United Kingdom

## Abstract

Expansion microscopy is a powerful technique for achieving nanoscale resolution, but labelling and detection using fluorescent proteins (FPs) have been hampered by signal degradation during expansion. Current approaches therefore rely heavily on immunostaining, ssDNA-FISH or HALO/SNAP-based detection systems. Here we show that substituting tyrosine with 3-iodotyrosine in the chromophore of superfolder GFP dramatically improves signal retention during expansion microscopy in zebrafish embryos. This enhancement specifically protects FPs during the formaldehyde-dependent anchoring step, and may additionally reflect better tolerance of the prolonged acidic conditions encountered during anchoring. 3-iodotyrosine-modified FPs open a door to reliable protein-based expansion microscopy.

**Figure 1. Investigating the application of 3-iodotyrosine-modified fluorescent proteins in expansion microscopy:** (A-B) Excitation (A) and emission (B) spectra of sfGFP with the indicated amino acids substituted in position 66 show increasing wavelength peaks as residue size increases. (C-D) Fluorescence measurement of protein heated at 95°C in ExM Denaturation Buffer for different times reveals that sfGFP(Y66IY) has stronger fluorescence after heating than any other tested protein. Proteins tested: (C) sfGFP with different halotyrosines in position 66, plus hyperfolder YFP (hfYFP); (D) wild-type and 3-iodotyrosine-bearing versions of several green fluorescent proteins. (E-F) Emission (E) and excitation (F) spectra of sfGFP(Y66IY) in PBS before and after 5 minutes at 95°C, as well as in 0.1 M NaOH. sfGFP(Y66IY) gains new fluorescence peaks after denaturation. (G-H) LifeAct-sfGFP(Y66IY) zebrafish embryos have improved fluorescence survival during ProExM. (G) Representative ProExM images of DAPI-stained zebrafish embryos injected with LifeAct-sfGFP(Y66IY) or LifeAct-sfGFP (n = 20). 4-fold expansion was achieved. Scale bar is 500 µm. (H) Quantification of zebrafish embryo LifeAct intensities from G (n = 20). (I-J) Fluorescence measurements of protein solutions show that sfGFP(Y66IY) fluorescence survives incubation in 1.4% formaldehyde better than sfGFP. (I) sfGFP(Y66IY) and sfGFP incubated for 16h at 37°C with different ProExM anchoring solution components. (J) Without 16h incubation at 37°C, the effect of 1.4% formaldehyde on sfGFP fluorescence is much milder. (K) Fluorescence measurement of protein in different pH 50 mM Tris buffers shows that sfGFP(Y66IY) is more resistant to acidic conditions than sfGFP. (L) Quantification of LifeAct fluorescence intensity in ProExM zebrafish embryos injected with LifeAct-hfYFP(Y67IY) or LifeAct-hfYFP, shows increased fluorescence in hfYFP(Y67IY) embryos (n = 9). All error bars show standard deviation. All experiments n = 3 unless otherwise stated.

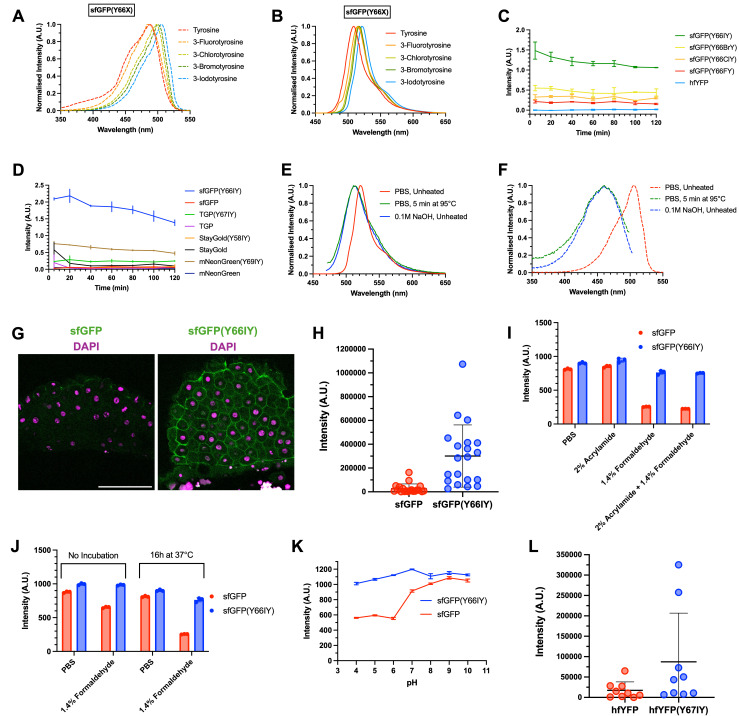



## Description

Expansion microscopy (ExM) overcomes microscopy resolution limits by isotropically increasing the sample size for detailed visualisation (Chen et al. 2015). The sample is embedded in a swellable hydrogel and grown many times its original size. Successful ExM requires breakdown of bonds in the sample, for which there are two common strategies: heat denaturation (Gambarotto et al. 2019) or proteinase K digestion (Tillberg et al. 2016). For samples with fluorescent proteins (FPs), both methods lose large amounts of signal during processing due to probe instability, resulting in weak fluorescence post-expansion (Tillberg et al. 2016). Instead most ExM methodologies rely on post-expansion staining with nanobodies, antibodies, ssDNA-FISH or dyes (Wen et al. 2023). FPs with improved stability could facilitate clearer ExM visualisation, faster protocols, decreased labour requirement, better pre- and post-expansion comparisons, and reduced labelling error.

Proteins can be given new characteristics through non-canonical amino acid mutagenesis via genetic code expansion. GFP has been reported to get colour-shifted when tyrosine-66 in its fluorophore tripeptide is substituted with halotyrosines (Young et al. 2011). The excitation and emission peaks get larger as the halide size increases ( Fig. 1A- B), likely due to increased atomic size affecting fluorophore geometry. We observed that 3-iodotyrosine (IY)-bearing superfolder GFP (sfGFP(Y66IY)) has a yellow band visible to the naked eye without illumination on SDS-PAGE gels, despite a prior 5-minute 95°C step that ordinarily causes loss in fluorescence. We hypothesised that sfGFP(Y66IY) may have improved thermostability and be useful for heat denaturation ExM.

Purified proteins were heated at 95°C for different times in ExM Denaturation Buffer and their fluorescence measured. We found that sfGFP(Y66IY) has stronger post-heating fluorescence than variants bearing smaller halotyrosines ( Fig. 1C ), namely 3-fluorotyrosine (FY), 3-chlorotyrosine (ClY), and 3-bromotyrosine (BrY). sfGFP(Y66IY) was also brighter than hyperfolder YFP (hfYFP), which is considered one of the best probes for ExM (Campbell et al. 2022). There is however a large drop in fluorescence intensity (~500-fold) in comparison to unheated sfGFP(Y66IY). It produced drastically higher post-heating fluorescence than wild-type sfGFP ( Fig. 1D ), as well as wild-type and IY-bearing versions of Thermostable Green Protein (TGP) (Close et al. 2015), StayGold (Hirano et al. 2022), and mNeonGreen (Shaner et al. 2013). The IY-bearing versions of TGP and mNeonGreen showed stronger post-heating fluorescence than wild-type counterparts, while StayGold(Y58IY) was non-fluorescent. We speculate that the specific TYG tripeptide fluorophore of sfGFP may give it a chemical advantage over the QYG fluorophore of TGP and the GYG fluorophores of StayGold and mNeonGreen when IY is substituted in (Pédelacq et al. 2006; Shaner et al. 2013; Close et al. 2015; Hirano et al. 2022). The quantum yield of sfGFP(Y66IY) was determined to be 0.84, indicating higher photon efficiency than the 0.65 of sfGFP (Pédelacq et al. 2006). Spectra of sfGFP(Y66IY) before and after 5 minutes at 95°C showed a change in fluorescence peaks, shifting from a 505 nm excitation peak to 459 nm ( Fig. 1E ), and from a 522 nm emission peak to 512 nm (Fig.1F). When denatured in 0.1M NaOH, fluorescent properties are observed with the same peaks as the heated sample. Rather than being traditionally thermostable, in which the protein keeps its structure in high temperature, this suggests sfGFP(Y66IY) is fluorescent when denatured.

We tested whether sfGFP(Y66IY) would be useful in heat denaturation ExM, expanding 1000-cell stage zebrafish embryos injected with LifeAct fusion proteins of sfGFP and sfGFP(Y66IY). No signal was detected from either protein, likely due to the large drop in sfGFP(Y66IY) intensity observed between unheated and heated samples. When we tested the proteins in proteinase K digestion ExM (ProExM), we were surprised to find that sfGFP(Y66IY) embryos were tremendously brighter than sfGFP embryos post-expansion ( Fig. 1G- H). To identify the mechanism behind this, we measured purified protein fluorescence in the presence of different ExM components, and only saw a difference between sfGFP(Y66IY) and sfGFP when testing the anchoring step in 2% acrylamide and 1.4% formaldehyde carried out prior to gelation. We identified that when incubated in formaldehyde solution for 16h at 37°C, sfGFP loses considerable fluorescence but sfGFP(Y66IY) does not ( Fig. 1I ). This does not happen without the incubation step ( Fig. 1J ). Formaldehyde solutions are neutral, but can become acidic over time through oxidation into formic acid.(Fox et al. 1985) We measured fluorescence of sfGFP(Y66IY) and sfGFP at different pHs and observed that sfGFP(Y66IY) is more stable under acidic conditions than sfGFP ( Fig. 1K ). We propose that sfGFP(Y66IY) is superior in ProExM because it retains its fluorescence when the anchoring solution becomes acidic during incubation, while sfGFP does not.

To find out if substituting IY into other FPs also improves their applicability in ProExM, we injected purified LifeAct fusion proteins of hfYFP and hfYFP(Y67IY) into zebrafish embryos and carried out ProExM. We found that hfYFP(Y67IY) embryos had increased LifeAct fluorescence than those with hfYFP (Fig.1L), although not as extreme an improvement as seen for sfGFP. This suggests that the approach of substituting IY into FPs to improve fluorescence in ProExM is translatable between FPs. Our work shows that sfGFP(Y66IY) is more fluorescent than other FPs after high temperature and that IY-bearing FPs are more tolerant of acidic conditions. IY-bearing FPs give improved signal in ProExM than their wild-type counterparts, increasing the convenience of ExM through the removal of the need for post-expansion staining, which often introduces labelling error. For further tool development, application of acid-resistant FPs without non-canonical amino acids should be investigated.

## Methods

**Protein Purification**

All proteins were cloned via Gibson assembly into pETMCN vectors with N-terminal 10xHis-tags for expression in BL21(DE3) bacteria. Protein purification was carried out as in Ivorra-Molla et al. (2024), except for those with non-canonical amino acids, which were carried out as in Scott et al. (2025), with 2 mM of the relevant amino acid used instead of 3-aminotyrosine and pTYR(MjIYRS2-1(D286)-MjR1X3) plasmid (a gift from Kensuko Sakomoto’s lab) used as the bacterial tRNA synthetase and tRNA plasmid. This plasmid constitutively expresses the necessary genetic code expansion components, so arabinose induction was not needed. Protein concentrations were determined by Bradford assay and nanodrop spectrophotometry.

**Fluorescence Measurement**

Fluorescence measurement of purified proteins in solution was carried out using black 96-well plates with 100 µL solution per well in a Varioskan Flash plate reader (Thermo Scientific). Samples were excited at 488 nm, and emissions were collected between 510-550 nm with a step-size of 1 nm. Maxima values were taken from each reading for analysis. Three repeats were done of each and means calculated, as well as a buffer only background control, the mean of which was subtracted from each sample. 5 µM purified protein was used in all experiments. Collection of excitation and emission spectra and calculation of quantum yield were carried out as in Ivorra-Molla et al. (2024). Spectra were normalised against the highest mean intensity in each dataset.

**Zebrafish Embryo Microinjections**

Wild-type zebrafish were maintained at 28.5°C with a 10/14h light/dark cycle, in accordance with UK Home Office guidelines and University of Warwick animal welfare regulations. One-cell stage embryos were microinjected with 2000 pg protein in 20 mM HEPES 150 mM NaCl pH 7.4. Following injection, embryos were grown at 28.5°C until 1000-cell stage (3h). Embryos were fixed in 4% formaldehyde with 4% sucrose overnight at 4°C, then washed in PBS and manually dechorionated.

**Heat Denaturation ExM**

The protocol used in Gambarotto et al. (2019) was adapted for zebrafish embryos. Briefly, fixed embryos were anchored at 37°C for 16h with 350 rpm shaking in freshly prepared 1.5 mL Anchoring Solution (2% acrylamide and 1.4% formaldehyde in PBS). Embryos were seeded on coverslips and gelation was carried out with the following recipe: PBS with 19% sodium acrylate, 10% acrylamide, 0.1% N,N’-methylenebisacrylamide, 0.5% ammonium persulfate, 0.5% tetramethylethylenediamine. The gelation was allowed to continue in a humidity chamber with a wet tissue at 37°C for 2h. The samples were transferred to tubes of 1 mL Denaturation Buffer (50 mM Tris pH 9, 200 mM NaCl, 200 mM SDS). These were heated at 95°C for 1.5h, and then aspirated. The samples were washed with PBS four times, 10 minutes with rotation each time. The samples were stained with DAPI (2 µg/mL in PBS) for 20 minutes with rotation at room temperature, then washed three times with PBS. The gels were removed from the coverslips and each expanded in 10 mL ddH _2_ O overnight and washed for 20 minutes in fresh ddH _2_ O, which was replaced again for imaging.

**ProExM**

The protocol used in Tillberg et al. (2016) was adapted for zebrafish embryos. Briefly, anchoring and gelation of fixed zebrafish embryos were carried out as before. Samples were incubated in digestion solution (50 mM Tris pH 8, 1 mM EDTA, 0.5% Triton X-100, 1 M NaCl) with 200 µg/mL proteinase K for 4h at 37°C. Gels were then stained with DAPI and expanded as before.

**Microscopy**

Expanded embryos were imaged on an Olympus FVMPE-RS multiphoton microscope with a 10X air objective. Analysis was performed using FIJI. Actin-associated signal (localised to plasma membranes) was segmented from cytoplasmic signal using the Trainable Weka Segmentation plugin (Arganda-Carreras et al. 2017). Segmented images were used to measure fluorescent signal localised to membranes and cytoplasm. Total actin-associated signal, accounting for how much actin is labelled, was calculated by subtracting the cytoplasmic signal from the actin-associated signal and multiplying by the area of the actin-associated signal.

## Reagents

3-iodotyrosine, #I8250, MilliporeSigma

3-bromotyrosine, #OR1010936, Apollo Scientific

3-chlorotyrosine, #512443, Sigma-Aldrich

3-fluorotyrosine, #AB169176, ABCR
